# Impact of Nasal Anatomical Variation Subtype on Surgical Outcomes for Rhinogenic Contact Point Headache

**DOI:** 10.3390/diagnostics15020121

**Published:** 2025-01-07

**Authors:** Salvatore Lavalle, Annalisa Pace, Giuseppe Magliulo, Mario Lentini, Jerome Rene Lechien, Christian Calvo-Henriquez, Federica Maria Parisi, Giannicola Iannella, Antonino Maniaci, Daniela Messineo

**Affiliations:** 1 Department of Medicine and Surgery, University of Enna “Kore”, 94100 Enna, Italy; salvatore.lavalle@unikore.it (S.L.); or mario.lentini@asp.rg.it (M.L.); 2 Department of ‘Organi di Senso’, University “Sapienza”, Viale Dell’università, 33, 00185 Rome, Italy; annalisa.pace@uniroma1.it (A.P.); giuseppe.magliulo@uniroma1.it (G.M.); giannicola.iannella@uniroma1.it (G.I.); 3 Rhinology Study Group of the Young Otolaryngologists-International Federation of Otorhinolaryngological Societies, 91190 Paris, France; jerome.lechien@unimons.ac.be (J.R.L.); christian.calvo.henriquez@gmail.com (C.C.-H.); federicamariaparisi1@gmail.com (F.M.P.); 4 ASP Ragusa-Hospital Giovanni Paolo II, 97100 Ragusa, Italy; 5 Department of Otolaryngology-Head and Neck Surgery, Faculty of Medicine, UMONS Research Institute for Health Sciences and Technology, University of Mons (UMons), 7000 Mons, Belgium; 6 Department of Otorhinolaryngology-Head and Neck Surgery, Hospital Complex of Santiago de Compostela, 15706 Santiago de Compostela, Spain; 7 Department of Medical, Surgical Sciences and Advanced Technologies G.F. Ingrassia, 95123 Catania, Italy; 8 Department of Radiological Sciences, Oncology and Anatomo-Pathological Science, “Sapienza” University of Rome, 00184 Rome, Italy; daniela.messineo@uniroma1.it

**Keywords:** rhinogenic contact point headache, endoscopic sinus surgery, nasal anomalies, CT scan, surgical outcomes

## Abstract

**Background/Objectives:** Rhinogenic contact point headache (RCPH) is a controversial secondary headache disorder involving mucosal contact points in the nasal sinuses. The efficacy of surgical versus medical management has been debated, with some studies showing excellent long-term outcomes but others citing placebo effects. This study aimed to clarify the correlation with nasal anatomical variation detected by CT and RCPH treatment outcomes. **Methods:** A prospective cohort study was conducted on 90 RCPH patients undergoing surgery or medical therapy. Patients were diagnosed using CT scans, endoscopy, and lidocaine testing. The surgery group had endoscopic procedures to remove contact points. The medical group received intranasal steroids. Outcomes were measured by visual analog scale (VAS) for pain and headache frequency. Predictors like age, gender, and nasal anatomical variations were analyzed. **Results:** The surgery group showed significant reductions in VAS scores (6.02 to 2.51, *p* < 0.001) and headache frequency (9.11 to 3.04, *p* < 0.001). The medical group did not improve significantly. All nasal subtypes improved with surgery but concha bullosa had worse VAS outcomes (4.0) than septal deviation (1.8, *p* < 0.001) or spur (1.73, *p* < 0.001). Multivariate analysis found nasal anomalies predicted postoperative VAS scores (*p* < 0.001) but not headache frequency (*p* = 0.255). **Conclusions:** Surgery demonstrated superiority over medications for RCPH. This study provides new evidence that preoperative CT scans should be considered a non-invasive gold standard for analyzing nasal subtypes as they significantly influence surgical success, with concha bullosa associated with worse pain relief. Larger studies should validate these findings to optimize RCPH management.

## 1. Introduction

Rhinogenic contact point headache (RCPH) is considered a secondary form of headache disorders according to the International Headache Society guidelines [1]. It is now completely accepted that intranasal contact points between two opposing intranasal mucosal surfaces, such as the nasal septum and lateral wall of the nose, can be a cause of headache and facial pain [2]. RCPH is characterized by headaches in the absence of inflammatory signs, hyperplastic mucosa, purulent discharge, sinonasal polyps, or tumors [3]. However, the underlying pathogenesis remains debated, particularly regarding diagnostic approaches and treatment options [4]. There are multiple anatomical situations that may lead to rhinogenic contact point headache such as septal spurs or middle turbinate disorders, such as hypertrophic, deformed, or hyperpneumatized (concha bullosa), in the absence of inflammation of nasal mucosa [5]. RCHP is quickly detectable and quantified by sinonasal endoscopy or computed tomography (CT) [6]. CT scans allow for detailed visualization of the nasal anatomy, facilitating the identification of potential structural abnormalities, which may contribute to contact points leading to headaches [7,8].

It has also been suggested that endoscopic sinus surgery may be a potential strategy in the management of symptoms of RCPH, as assessed by VAS and MIDAS scoring [9]. Surgical interventions have promising results, but the field remains divided about their long-term efficacy and the possible role of placebo effects. A recent systematic review analyzed data from a variety of studies that have compared the effectiveness of surgical versus medical management of RCPH, both in the short and long term [10,11]. Cantone et al. reported improvement in VAS headache scores for 53 patients at 6 months post-endoscopic surgery [12], and Guyuron et al. demonstrated excellent results with septoplasty for migraine frequency, duration, and intensity in 89/100 patients at 5-year follow-up [13]. However, some of these positive results continue to be debated within medical circles due to critics generally holding the belief that this may be due to the placebo effect and cognitive dissonance rather than because of the surgical intervention. According to some researchers, this reduction can become temporary (<2 years) due to a changed subjective perception due to cognitive dissonance. West et al. suggested that the success of surgery may be confined to specific subgroups of patients, improvement in symptoms being a consequence of neuroplasticity or cognitive adaptation rather than a direct anatomic correction per se [14]. Such divergent views call for a more serious study on the mechanism underlying surgical success in the treatment of RCPH.

However, the current literature is lacking in evaluating how distinct nasal anatomical variables affect the way that RCPH is treated. Assessing this association can yield insightful information that helps physicians customize the best course of action for each patient’s unique nasal pathology. The purposes of this prospective cohort study were to investigate the impact of CT nasal anatomical variation on improved outcomes following treatment. This prospective cohort study’s main goal was to find out how distinct nasal structural variations—found by CT imaging—affect the way patients with RCPH respond to treatment. We postulated that the specific mucosal contact point subtype—such as septal deviation, septal spur, or concha bullosa—may influence the effectiveness of endoscopic sinus surgery or medical therapy in enhancing quality of life. This study aims to offer crucial insights to help clinicians choose the best treatment plan for each patient based on their underlying sinonasal pathology by assessing the impact of these unique nasal anatomical anomalies.

## 2. Materials and Methods

### 2.1. Study Design and Ethics

**Figure 1:**
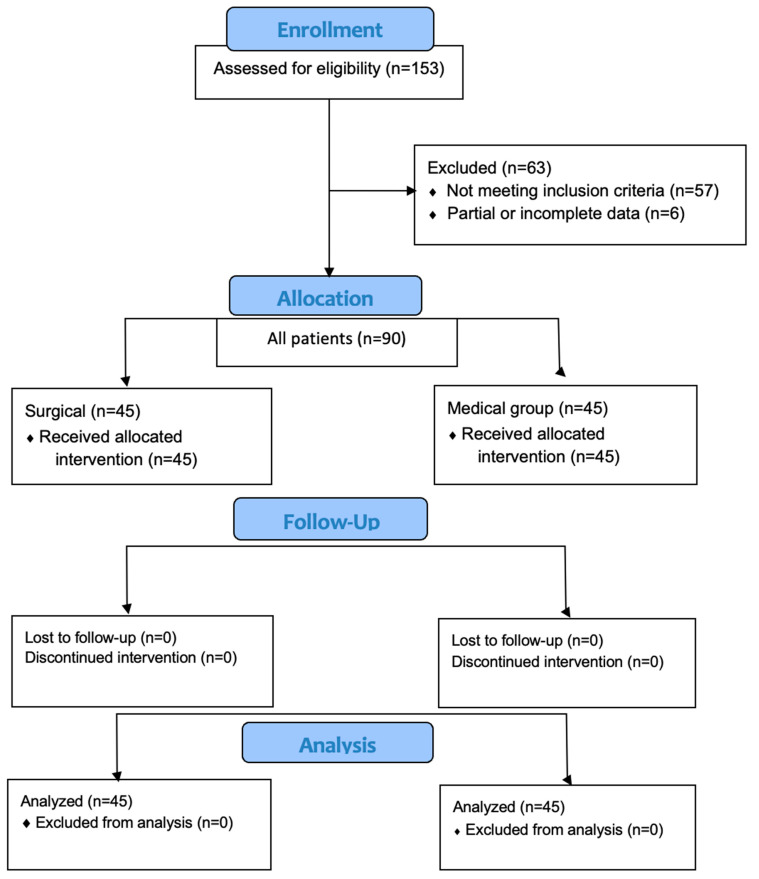
Flow diagram of the study protocol.

A prospective cohort study was conducted, adhering strictly to the recommendations of the Strengthening the Reporting of Observational Studies in Epidemiology (STROBE) guidelines [15]. The study’s workflow is depicted in Figure 1.

**Figure 2:**
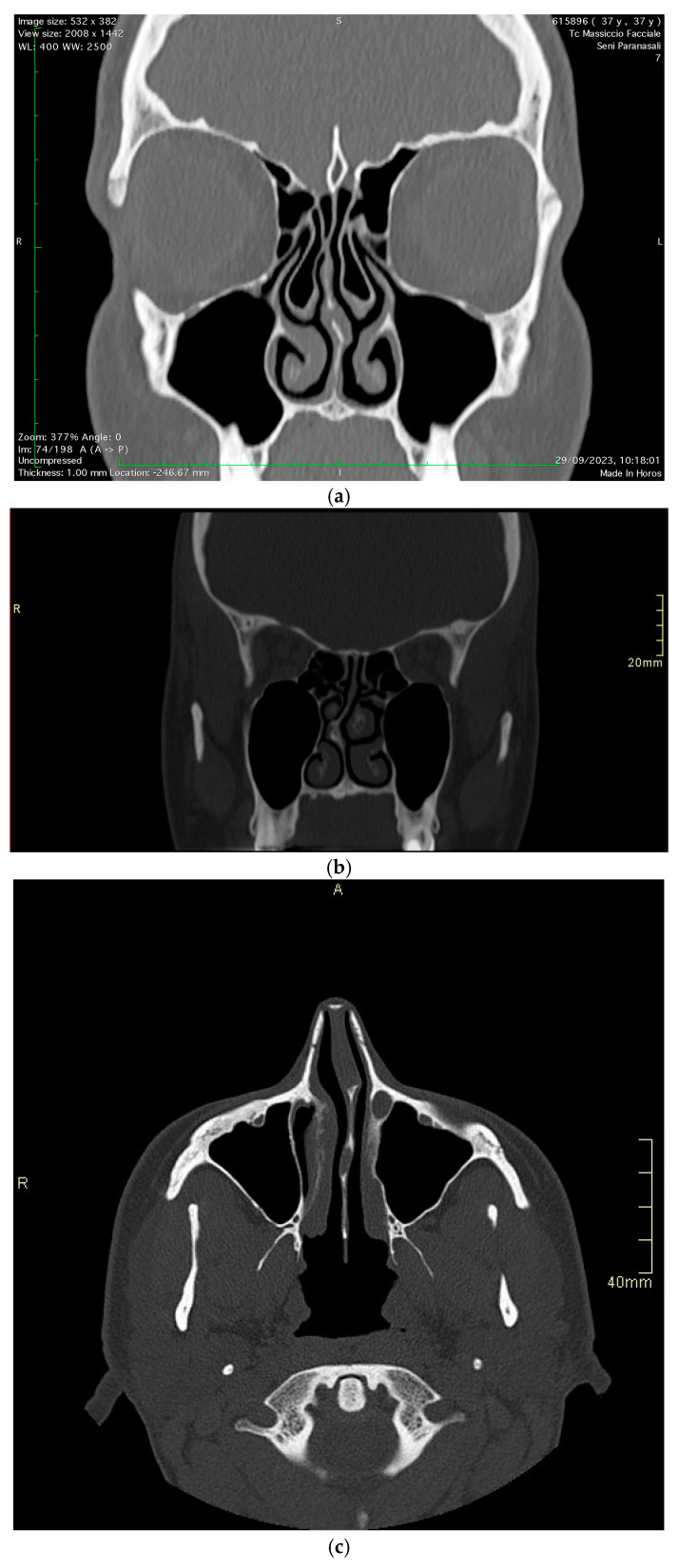
Different nasal structure anomalies. (**a**) Concha bullosa, (**b**) septal spur, (**c**) septal deviation.

The study encompassed a patient pool from January 2021 to June 2024, consisting of 90 patients with chronic headaches attributed to nasal structural anomalies (Figure 2a–c).

We followed a standardized protocol to allocate each patient to treatment groups:

1. Initial evaluation included CT scanning, nasal endoscopy, and lidocaine testing.
2. Patients meeting inclusion criteria were counseled about both surgical and medical treatment options.
3. Treatment allocation was based on the following:
  - Severity of anatomical variation as determined by CT imaging;
  - Patient preference after detailed discussion of risks and benefits;
  - Presence of significant functional nasal obstruction;
  - Prior response to medical therapy.
4. To ensure balanced representation, subjects were stratified by anatomical variation type (concha bullosa, septal deviation, septal spur) with equal distribution (15 patients per variation) in both surgical and medical groups.
5. Regular monitoring was established for both groups with identical follow-up schedules [2].

All eligible patients were included in two different treatment groups, the medical and surgical groups. In addition, an equal number of patients per anatomical variation were distributed in the medical and surgical group. We aimed as a primary objective to identify the impact of CT-detected nasal anatomical variations on treatment outcomes. Secondary objectives were the following: (1) the efficacy of surgical intervention versus medical management in treating RCPH; (2) the impact of each specific nasal anatomical subtype; (3) identify potential predictors of treatment outcomes. The University Ethics Committee granted approval for the study (approval code 24121-21/05/2021). All participants provided written informed consent after being briefed on the study’s aims and procedures, which conformed to the ethical standards laid out in the Declaration of Helsinki.

Inclusion and Exclusion Criteria

Patients were eligible for inclusion if they

1. Reported chronic headache or facial pain unresponsive to analgesics.
2. Had a confirmed diagnosis of septal deviation, septal spur, or concha bullosa.
3. Were aged 20 years or above.
4. Exhibited a positive response to a lidocaine test, where pain relief was achieved upon lidocaine application to the nasal cavity.
5. Were undergoing medical or surgical treatment for RCPH.

Exclusion criteria were as follows:

1. Less than one year of consecutive clinical and diagnostic follow-up.
2. Presence of comorbid conditions including allergies, other sinonasal disorders, migraines, cluster headaches, ophthalmologic or vascular disorders, hypertension, pregnancy, or temporomandibular joint disorders.
3. A history of previous sinonasal surgeries.

### 2.2. Diagnostic Workup

All patients underwent an unenhanced sinus CT examination. The studies were performed on a 64-MDCT (LightSpeed VCT 64 Channel; GE, Milwaukee, USA) scanner with an FOV of 14–16 cm and a slice thickness of 0.625 mm. The axial plane utilized was the inferior orbital meatal plane, also known as the anthropologic plane. After image acquisition, postprocessing was performed to generate coronal and sagittal reconstructions. The CT scans were anonymized and examined independently by two experienced head and neck radiologists with more than 5 years of experience, blinded to clinical information. The CT scans were evaluated for the presence of anatomic variants of the sinonasal cavities. Nasal septum deviation (NSD) was assessed by measuring the angle between the crista galli to maxillary crest line and the line to the maximum deviation of the nasal septum. Pathological deviation was defined as an angle >5° [16]. A septal spur (SS) was identified as a bony protrusion of the nasal septum in the coronal and axial CT sections. Concha bullosa (CB) was recognized in the coronal and axial CT sections, appearing as an air-filled space within the middle meatus, enclosed by an ovoid bony rim. Two independent ENT specialists (A.M and S.L) conducted clinical examinations and fiberoptic nasal endoscopies using rigid endoscopes (2.7 or 4 mm, Storz, Tuttingen, Germany) to identify mucosal contact points. and were corroborated by CT scans with multiplanar reconstructions. A confirmatory lidocaine test was administered during an acute headache episode. A cotton ball saturated with a 5% lidocaine solution was placed for 15 min, with a reduction in pain intensity of more than 50% considered a positive response. Pain levels were measured using a ten-point VAS, with endpoints labeled ‘no pain’ and ‘maximum pain’. Age and gender were analyzed as potential effect modifiers.

### 2.3. Treatment and Interventions

Patients were assigned into two groups based on the established protocols:

- Group A (surgical intervention): patients underwent endoscopic surgical procedures by the same surgeons (A.M., M.L.), focusing on the removal of mucosal contact points, lateral resection of concha bullosa, and septoplasty or submucous resection for septal deviations and spurs.
- Group B (medical comparison): Patients received non-surgical treatment consisting of a standardized treatment consisting of intranasal fluticasone propionate, 125 mg per dose, with two sprays in each nostril every morning for 15-day cycles each month, without addressing contact points surgically. The patients were given detailed written instructions on how to take the medication properly, thus guaranteeing uniformity of delivery.

For quality control and consistency, all procedures were adequately documented using standardized surgical reports and photographic documentation. The surgical team met regularly to ensure that the technique and approach were the same for all cases, to make sure that the outcomes would be reliable and comparable among all study participants.

### 2.4. Surgical Protocol and Postoperative Care

The surgical protocol began with extensive preoperative preparation, including detailed CT reviews in preparation for surgery. All the procedures were performed under general anesthesia. The specific surgical techniques were adapted to each anatomic variation while following the same standardized approaches. The surgical treatment consisted of a lateral lamella resection with the preservation of medial lamella in concha bullosa cases while preserving much care to preserve mucosa. An empty nose syndrome was avoided by performing the procedure with control over how much resection should be made. Septal deviation cases received traditional Cottle’s septoplasty technique with the preservation of at least 1.5 cm of L-strut. This involved the elevation of bilateral mucoperichondrial flaps and selective reshaping, or removal of cartilage as required. In cases of septal spurs, our approach was the selective removal of the bony or cartilaginous spur using mucosal preservative techniques with endoscopic confirmation of the elimination of the contact point. Standardized care postoperatively for each case was conducted regarding silastic splinting for seven days postoperatively, nasal irrigations beginning 24 h post-surgery, and weekly endoscopic follow-ups in the first postoperative month. Pain was also managed on a standard continuous scale.

### 2.5. Statistical Analysis

Sample size calculation was made by G-Power 3.1.9.7 software. Considering previous studies investigating RCPH surgical outcomes [10,17,18,19], we expected a moderate effect size (Cohen’s d = 0.6) when comparing pre- and post-treatment VAS scores between surgical and medical groups. With a two-tailed α of 0.05 and desired power (1-β) of 0.85, the minimum required sample size was calculated as 41 patients per group. Assuming a 10% dropout rate and equal distribution according to the three anatomical variations, we added to our target enrollment 45 patients per group, for a total sample of 90 patients, 15 patients per anatomical variation subtype in each group. This sample size was similarly sufficient to detect clinically important changes in secondary outcomes, such as headache frequency and MIDAS scores, based on effect sizes from previously reported studies in similar studies [12,13].

Descriptive statistics were summarized as means ± standard deviations or proportions. Paired sample t-tests were applied to normally distributed continuous variables to compare pre- and post-treatment observations. The Mann–Whitney U test was utilized for continuous variables that were not normally distributed. To evaluate the effect of different independent variables, such as age, sex, preoperative RCPH scores, and specific anatomical abnormalities, on the VAS outcomes post-treatment, a Kruskal–Wallis test was performed. This non-parametric test assessed the variance in surgery-driven outcomes across the different patient subgroups. The analysis was conducted using IBM SPSS Statistics for Windows (SPSS, Version 29.0, IBM Corp., Armonk, NY, USA).

## 3. Results

### 3.1. Demographic Features

All patients with RCPH underwent CT examination. A total of 90 patients were included in the study, including 30 for each of the three anatomical variations detected. As summarized in Table 1, there was a mean age of 38.87 ± 4.30 years, where 40% were male and 60% were female (Table 1).

Female patients (n = 54) presented with more severe baseline symptoms (mean VAS 7.8 ± 1.1) than males (6.9 ± 1.2, *p* = 0.031). In addition, the age distribution showed that the majority of patients (58%) were between 30 and 45 years old.

### 3.2. Surgical vs. Medical Outcomes

**Figure 3:**
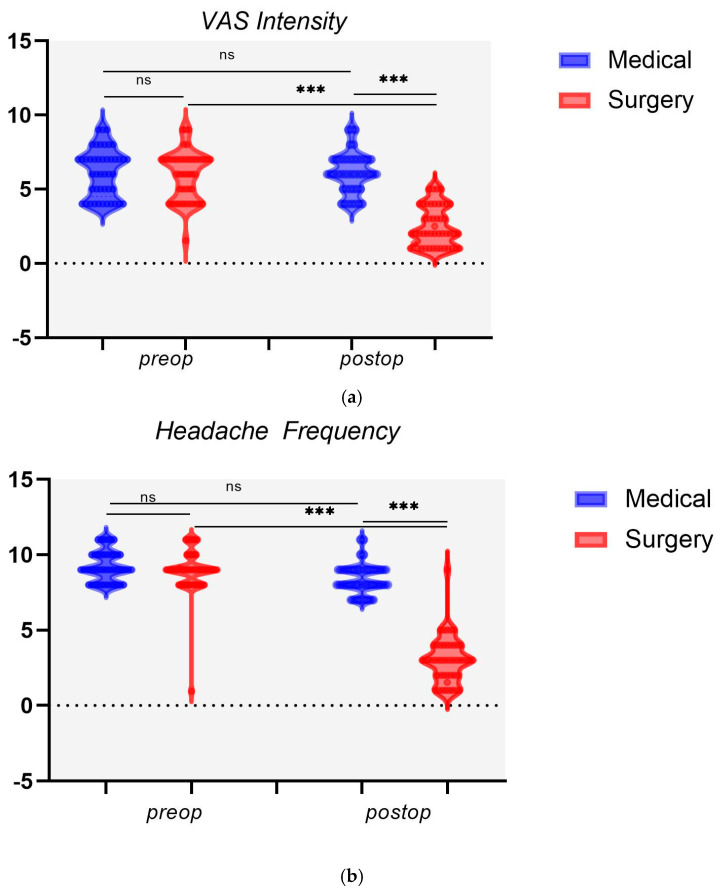
Surgical vs. medical RCPH outcomes. Abbreviation: RCPH, rhinogenic contact point headache. (**a**) VAS intensity. (**b**) Headache frequency. Abbreviations: ns, not significant; *** *p* < 0.001.

The surgery group experienced a significant reduction in VAS intensity scores postoperatively (mean reduction from 6.02 ± 1.54 to 2.51 ± 1.34, *p* < 0.001) as compared to the medical group, which paradoxically showed an increase (Table 1). The mean headache frequency for the surgery group was significantly reduced from 9.11 ± 0.96 preoperatively to 3.04 ± 1.54 postoperatively (*p* < 0.001). Conversely, the medical group did not experience a significant change, with the mean headache frequency slightly decreasing (9.22 ± 0.99 vs. 8.36 ± 0.96; *p* < 0.001) (Figure 3).

There was a noteworthy postoperative improvement in the MIDAS grade percentage for the surgery group, with a significant increase in lower grade I-II from 24.44% to 53.33% (*p* < 0.001). Conversely, the medical group showed no significant change in MIDAS grade at follow-up, with a slight improvement from 20% to 15.56% of the cases.

Age subgroup analyses according to years < or >35 were not associated with a significant treatment response in both the medical or surgical groups (*p* = 0.126 and 0 = 324, respectively).

### 3.3. Nasal Variation Outcomes

**Figure 4:**
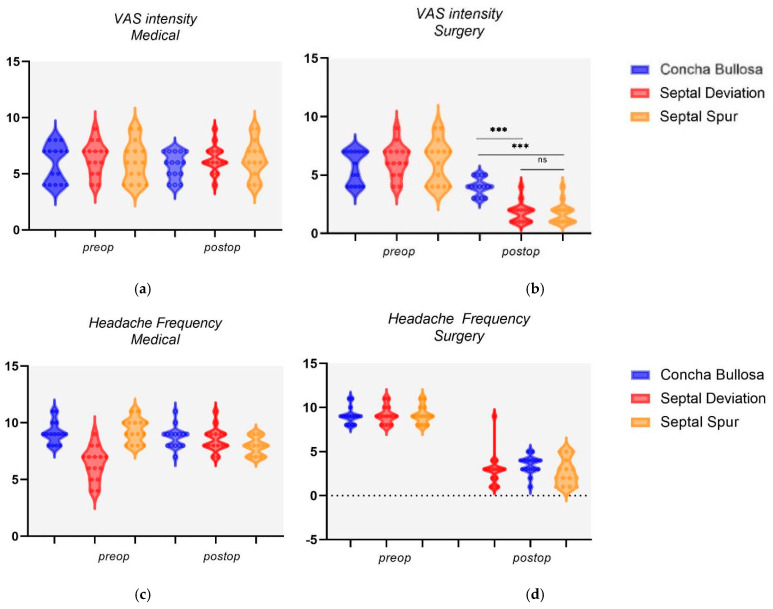
(**a**–**d**) Subgroup outcomes according to nasal anomalies. (**a**) VAS intensity in medical group; (**b**) VAS intensity in surgical group; (**c**) headache frequency in medical group; (**d**) headache frequency in surgical group. *** *p* < 0.001; ns, not significant.

In the intragroup analysis for the three nasal anomalies, surgical intervention led to significant improvements in both VAS intensity and headache frequency at follow-up (Table 2). Conversely, the medical treatment did not result in a significant change in VAS intensity for pain across the three nasal pathologies studied. However, there was a statistically significant reduction in headache frequency post-treatment for patients with NSD (9.2 ± 0.97 vs. 8.53 ± 1.02; *p* = 0.045) and SS (9.33 ± 1.01 vs. 7.93 ± 0.77; *p* = 0.001), with the latter group experiencing the most notable improvement. Instead, patients with CB reported a marginal decrease from a preoperative value of (9.13 ± 0.95 vs. 8.73 ± 0.92; *p* = 0.082). In the intergroup analysis for nasal anomalies, those with CB reported a VAS intensity of 4 ± 0.73 after surgery, whereas the NSD group reported a significantly lower VAS intensity of 1.8 ± 0.83 (*p* < 0.001) (Figure 4a–d).

In addition, when comparing postoperative VAS intensity between patients with CB and those with SS who underwent surgery, a similar pattern emerges (4 ± 0.73 vs. 1.73 ± 0.85; *p* < 0.001). In contrast, medical management showed no significant differences in postoperative VAS intensity between the groups, indicating that medical treatment did not significantly alter the VAS intensity for pain relief between these patient groups. For the CB and NSD comparison, the VAS intensity post-treatment was 5.73 ± 1.12 and 6.33 ± 1.19, respectively (*p* = 0.166). Similarly, for the CB and SS comparison, the VAS intensity post-treatment was 5.73 ± 1.12 and 6.26 ± 1.56, respectively (*p* = 0.294).

### 3.4. Predictors of Treatment Outcome

Our multivariate analysis sought to pinpoint predictive factors for surgical outcomes in RCPH patients, focusing on VAS intensity and headache frequency post-surgery (Table 3).

Notably, diagnosing nasal anomalies emerged as a significant factor associated with postoperative VAS intensity scores (F = 30.909, *p* < 0.001). However, the presence of a nasal anomaly did not correlate significantly with headache frequency post-surgery (F = 1.330, *p* = 0.255). Additionally, the study found that age does not significantly impact surgical outcomes, as evidenced by its lack of influence on postoperative VAS scores (F = 0.036, *p* = 0.850) and headache frequency (F = 0.043, *p* = 0.914). Similarly, gender did not significantly affect postoperative VAS scores (F = 1.271, *p* = 0.266) or headache frequency (F = 2.333, *p* = 0.134). The initial pain levels did not predict the success of surgery both for postoperative VAS outcomes (F = 1.378, *p* = 0.247) or headache frequency (F = 0.940, *p* = 0.338). On the other hand, a higher frequency of headaches before surgery was significantly linked to increased postoperative VAS scores (F = 4.756, *p* = 0.035). Yet, this did not significantly predict postoperative headache frequency (F = 0.155, *p* = 0.696). Lastly, the MIDAS score’s predictive value for postoperative outcomes was not significant for VAS scores (F = 1.378, *p* = 0.247) or headache frequency (F = 1.487, *p* = 0.229).

## 4. Discussion

*RCPH*, characterized by mucosal contact points in nasal sinuses, presents a debated landscape in terms of optimal management [11,17]. Our study, focusing on nasal anatomical variations and their impact on treatment outcomes, investigated the efficacy of surgical intervention versus medical therapy in RCPH. Consistent with previous studies [18,19], we observed a significant reduction in VAS intensity scores postoperatively in the surgery group, indicating a decrease in pain severity (mean reduction from 6.02 ± 1.54 to 2.51 ± 1.34, *p* < 0.001). This aligns with the literature evidence, which reported similar reductions in pain intensity following surgical intervention for RCPH patients [5,10]. In contrast, the medical group in our study showed an increase in VAS intensity scores, indicating that medical therapy did not effectively alleviate pain. This finding is consistent with the study conducted by Behin et al., which demonstrated that medical treatment alone may not provide significant pain relief for RCPH patients [20]. These results highlight the potential superiority of surgical intervention over medical therapy in terms of pain management for RCPH. Regarding headache frequency, our study demonstrated a significant reduction in the surgery group from 9.11 ± 0.96 preoperatively to 3.04 ± 1.54 postoperatively (*p* < 0.001). This aligns with studies by Bektas et al., which reported similar decreases in headache frequency following surgical treatment for RCPH patients [9]. In contrast, the medical group in our study did not show a significant change in headache frequency, which suggested that medical therapy may have limited efficacy in reducing headache frequency in RCPH patients. Our study is the first evidence found analyzing the impact of nasal subtypes on treatment outcomes, finding that surgical intervention led to significant improvements in both VAS intensity scores and headache frequency across the three nasal anomalies studied. Specifically, our results showed that patients with NSD and SS experienced the most notable improvement in headache frequency. However, patients with CB reported only a marginal decrease in headache frequency, suggesting worse outcomes for this specific subtype. Our multivariate analysis aimed to identify predictors of treatment outcomes in RCPH patients. Consistent with the previous literature [21], we found that nasal anomalies were significantly associated with postoperative VAS intensity scores (F = 30.909, *p* < 0.001), supporting the findings of our precedent study [10]. However, the presence of nasal anomalies did not significantly correlate with postoperative headache frequency. Additionally, our study corroborated previous evidence that age and gender do not significantly impact surgical outcomes in RCPH patients [22]. Long-term outcome stability remains debated. Some authors hypothesized temporary relief only from cognitive factors like dissonance, while others report long-lasting results [13]. Welge-Luessen et al. described excellent surgical outcomes at 112-month follow-up, with 65% overall improvement [23]. Our precedent analysis found that surgically treated patients reporting baseline VAS ≤ 4 or MIDAS ≤ 2 achieved better 3-year outcomes, while medically only VAS correlated (all *p* < 0.05) [10]. Our recent study confirmed at 1 y follow-up the predictive role of VAS intensity ≤ 4 but lower MIDAS grade was not a significant predictor. In this context, a CT scan provides a detailed and three-dimensional visualization of the nasal and sinus anatomy, allowing for the identification of structural nasal variations, and plays a crucial role in the treatment planning of RCPH. For instance, if an NSD or SS is identified, surgical intervention may be recommended to address the mucosal contact points contributing to headache symptoms. On the other hand, if CB is present, the surgical approach may need to be more nuanced due to the potential association with less effective pain relief. In this regard, it should be underlined that the significant relation between nasal anomaly subtypes and treatment outcomes was F = 30.909, *p* < 0.001. Although concha bullosa is very common, being an anatomic variant present in up to 20–30% of the general population, its relationship with the mechanisms of headache remains incompletely understood. The lower success rate of surgical intervention may be related to some of the following factors in these patients. Firstly, the pneumatized middle turbinate may affect the intranasal distribution of contact points beyond those reached by primary surgical intervention; secondly, the altered aerodynamics in concha bullosa patients might perpetuate mucosal inflammation or neurogenic stimulation even after the correction of other anatomical deformities.

It is important to recognize the study limitations, though. The comparatively modest sample size—just 90 individuals in total across the three nasal anatomical variation groups—is a significant drawback. Larger, multicenter trials would be required, according to the authors, to further validate their findings. The study’s single-center design restricts how broadly the findings may be applied. Furthermore, only patients with a minimum of one year of follow-up were included in the study. Despite the ongoing controversy regarding the durability of surgical advantages for RCPH, longer-term results beyond a year were not investigated. By extending the follow-up time, more information about the therapy effects’ long-term stability may become available. Despite the multivariate analysis’s identification of predictors of surgical outcomes, additional confounding variables might not have been taken into consideration. Further investigation into a wider variety of putative predictors, such as comorbidities and patient characteristics, may help clarify the critical factors influencing treatment outcomes for patients with RCPH.

## 5. Conclusions

In conclusion, CT can provide essential diagnostic information for accurate treatment planning and serves as a tool for guiding and evaluating surgical interventions. The integration of CT imaging in the management of RCPH enhances the precision of diagnosis and treatment, ultimately contributing to improved patient outcomes. It is important to note that our study has limitations, including its relatively small sample size and the need for larger multicenter studies to confirm our findings. Nonetheless, our results are consistent with the existing literature, supporting the superiority of surgical intervention over medical therapy in terms of pain intensity reduction and highlighting the influence of nasal anomalies on treatment outcomes. Future research should focus on conducting well-designed multicenter studies with larger sample sizes to further validate our findings and provide more robust evidence for the optimal management of RCPH and on the role of CT in directing the patient to the best treatment.

## Figures and Tables

**Table 1:** Baseline vs. treatment outcomes at follow-up. ^a^ *p* < 0.001 at intragroup analysis. ^b^ *p* < 0.001 at intergroup analysis.

		Preoperative				Postoperative			
		VAS Intensity	Head Frequency	MIDAS Grade%		VAS Intensity	Head Frequency	MIDAS Grade %	
**Surgery (*n* = 45)**	*mean*	6.02 ^a^	9.11 ^a^	*I–II*	24.44	2.51 ^a,b^	3.04 ^a,b^	*I–II*	53.33
	*sd*	1.54	0.96	*III–IV*	75.55	1.34	1.54	*III–IV*	46.67
**Medical (*n* = 45)**	*mean*	5.96	9.22	*I–II*	20	6.22	8.36	*I–II*	15.55
	*sd*	1.52	0.99	*III–IV*	80	1.33	0.96	*III–IV*	84.44

**Table 2:** Intergroup comparison according to nasal anomaly subtype.

VAS Intensity						
	Concha Bullosa	Septal Deviation	*p*-Value	Concha Bullosa	Septal Spur	*p*-Value
**Surgery**	4 ± 0.73	1.8 ± 0.83	*p* < 0.001	4 ± 0.73	1.73 ± 0.85	*p* < 0.001
**Medical**	5.73 ± 1.12	6.33 ± 1.19	0.166	5.73 ± 1.12	6.26 ± 1.56	0.294
**Headache Frequency**						
	**Concha Bullosa**	**Septal Deviation**	***p*-Value**	**Concha Bullosa**	**Septal Spur**	***p*-Value**
**Surgery**	3 ± 1.86	3.46 ± 1.02	0.408	3 ± 1.86	2.66 ± 1.44	0.580
**Medical**	8.73 ± 0.92	8.53 ± 1.02	0.577	8.73 ± 0.92	7.93 ± 0.77	0.015

**Table 3:** Multivariate analysis for postoperative VAS intensity and headache frequency outcomes after surgical treatment. Several preoperative variables were assessed. Abbreviations: preop, preoperative.

VAS Intensity			Headache Frequency	
Variable	F	Sig.	F	Sig.
Nasal anomaly subtype	30.909	<0.001	1.330	0.255
Age <>35 y	0.036	0.850	0.043	0.914
Sex M/F	1.271	0.266	2.333	0.134
VAS intensity preop <>4	1.378	0.247	0.940	0.338
Headache frequency preop <>9	4.756	0.035	0.155	0.696
MIDAS low–high grade	1.378	0.247	1.487	0.229

## Data Availability

Data are only available under authors’ request.

## References

[1] Nicholas M., Vlaeyen J.W.S., Rief W., Barke A., Aziz Q., Benoliel R., Cohen M., Evers S., Giamberardino M.A., Goebel A. (2019). The IASP classification of chronic pain for ICD-11: Chronic primary pain. Pain.

[2] Benoliel R., Svensson P., Evers S., Wang S.J., Barke A., Korwisi B., Rief W., Treede R.D., The IASP Taskforce for the Classification of Chronic Pain (2019). The IASP classification of chronic pain for ICD-11: Chronic secondary headache or orofacial pain. Pain.

[3] Headache Classification Subcommittee of the International Headache Society (2004). The international classification of headache disorders: 2nd edition. Cephalalgia.

[4] Herzallah I.R., Hamed M.A., Salem S.M., Suurna M.V. (2015). Mucosal contact points and paranasal sinus pneumatization: Does radiology predict headache causality?. Laryngoscope.

[5] Sollini G., Mazzola F., Iandelli A., Carobbio A., Barbieri A., Mora R., Peretti G. (2019). Sino-nasal anatomical variations in rhinogenic headache pathogenesis. J. Craniofac. Surg..

[6] Pasha R., Soleja R.Q., Ijaz M.N. (2014). Imaging for headache: What the otolaryngologist looks for. Otolaryngol. Clin. N. Am..

[7] Mariotti L.J., Setliff R.C., Ghaderi M., Voth S. (2009). Patient history and CT findings in predicting surgical outcomes for patients with rhinogenic headache. Ear Nose Throat J..

[8] Barinsky G.L., Hanba C., Svider P.F. (2020). Rhinogenic Headache in Children and Adolescents. Curr. Pain Headache Rep..

[9] Bektas D., Alioglu Z., Akyol N., Ural A., Abu-Samra M., Gawad O.A., Agha M. (2011). The outcomes for nasal contact point surgeries in patients with unsatisfactory response to chronic daily headache medications. Eur. Arch. Otorhinolaryngol..

[10] Maniaci A., Lechien J.R., Calvo-Henriquez C., Iannella G., Leigh S., Ingrassia A., Merlino F., Bannò V., Cocuzza S., La Mantia I. (2022). Long-term stability of outcomes of endoscopic surgery for rhinogenic contact point headache (Sluder’s neuralgia). Am. J. Otolaryngol..

[11] Maniaci A., Merlino F., Cocuzza S., Iannella G., Vicini C., Cammaroto G., Lechien J.R., Calvo-Henriquez C., La Mantia I. (2021). Endoscopic surgical treatment for rhinogenic contact point headache: Systematic review and meta-analysis. Eur. Arch. Otorhinolaryngol..

[12] Cantone E., Castagna G., Ferranti I., Cimmino M., Sicignano S., Rega F., Di Rubbo V., Iengo M. (2015). Concha bullosa related headache disability. Eur. Rev. Med. Pharmacol. Sci..

[13] Guyuron B., Kriegler J.S., Davis J., Amini S.B. (2011). Five-year outcome of surgical treatment of migraine headaches. Plast. Reconstr. Surg..

[14] West B., Jones N.S. (2001). Endoscopy-negative, computed tomography-negative facial pain in a nasal clinic. Laryngoscope.

[15] von Elm E., Altman D.G., Egger M., Pocock S.J., Gøtzsche P.C., Vandenbroucke J.P., STROBE Initiative (2014). The strengthening the reporting of observational studies in epidemiology (STROBE) statement: Guidelines for reporting observational studies. Int. J. Surg..

[16] Mohebbi A., Memari F., Mohebbi S. (2010). Endonasal endoscopic management of contact point headache and diagnostic criteria. Headache.

[17] Wee J.H., Lee J.E., Hong S.L., Shin J.M., Kim D.Y. (2015). Prospective study on the characteristics and postoperative improvement of rhinogenic headache. J. Rhinol..

[18] Bahadir O., Caylan R. (2011). Surgical outcomes for rhinogenic contact point headaches. Med. Princ. Pract..

[19] Behin F., Behin B., Bigal M.E., Lipton R.B. (2005). Surgical treatment of patients with refractory migraine headaches and intranasal contact points. Cephalalgia.

[20] Peric A., Rasic D., Grgurevic U. (2016). Surgical Treatment of Rhinogenic Contact Point Headache: An Experience from a Tertiary Care Hospital. Int. Arch. Otorhinolaryngol..

[21] Cocuzza S., Maniaci A., Di Luca M., La Mantia I., Grillo C., Spinato G., Motta G., Testa D., Ferlito S. (2020). Long-term results of nasal surgery: Comparison of mini-invasive turbinoplasty. J. Biol. Regul. Homeost. Agents.

[22] Altin F., Haci C., Alimoglu Y., Yilmaz S. (2019). Is septoplasty effective rhinogenic headache in patients with isolated contact point between inferior turbinate and septal spur?. Am. J. Otolaryngol..

[23] Welge-Luessen A., Hauser R., Schmid N., Kappos L., Probst R. (2003). Endonasal surgery for contact point headaches: A 10-year longitudinal study. Laryngoscope.

